# Quaternary Structure and Hetero-Oligomerization of Recombinant Human Small Heat Shock Protein HspB7 (cvHsp)

**DOI:** 10.3390/ijms22157777

**Published:** 2021-07-21

**Authors:** Lydia K. Muranova, Vladislav M. Shatov, Andrey V. Slushchev, Nikolai B. Gusev

**Affiliations:** Department of Biochemistry, School of Biology, Moscow State University, 19234 Moscow, Russia; lidiamuranova@gmail.com (L.K.M.); shatovm@inbox.ru (V.M.S.); sav_1414_fa@mail.ru (A.V.S.)

**Keywords:** small heat shock proteins, HspB7, cvHsp, oligomeric structure

## Abstract

In this study, a reliable and simple method of untagged recombinant human HspB7 preparation was developed. Recombinant HspB7 is presented in two oligomeric forms with an apparent molecular weight of 36 kDa (probably dimers) and oligomers with an apparent molecular weight of more than 600 kDa. By using hydrophobic and size-exclusion chromatography, we succeeded in preparation of HspB7 dimers. Mild oxidation promoted the formation of large oligomers, whereas the modification of Cys 126 by iodoacetamide prevented it. The deletion of the first 13 residues or deletion of the polySer motif (residues 17–29) also prevented the formation of large oligomers of HspB7. Cys-mutants of HspB6 and HspB8 containing a single-Cys residue in the central part of the β7 strand in a position homologous to that of Cys137 in HspB1 can be crosslinked to the wild-type HspB7 through a disulfide bond. Immobilized on monoclonal antibodies, the wild-type HspB6 interacted with the wild-type HspB7. We suppose that formation of heterodimers of HspB7 with HspB6 and HspB8 may be important for the functional activity of these small heat shock proteins.

## 1. Introduction

Small heat shock proteins (sHsps) form a large family of closely related proteins [1]. These proteins are expressed in practically all living beings including viruses, bacteria, plants, and animals [2,3,4]. sHsps form the first line of stress defense, protecting the cell from the accumulation of aggregates formed by denatured proteins [5]. Monomers of sHsp have a primary structure that can be divided into three parts. All sHsps have a conservative α-crystallin domain (ACD) containing 80–100 amino acid residues and located in the C-terminal part of the sHsp monomer. This domain is the hallmark of the sHsp family [6]. The ACD is flanked by difference in size and structure N-terminal domain (NTD) that often harbors sites of phosphorylation [7] and the comparably short C-terminal domain (CTD) that often contains conservative I/V-X-I/V motifs [8]. sHsp tends to form oligomers of variable sizes. Usually, the ACD containing several β-strands is responsible for the formation of stable dimers [9], the highly mobile CTD can participate in interdimer contact and the formation of small oligomers [10,11], and the NTD appears to be responsible for the formation of large sHsp oligomers [8,12]. The human genome contains ten genes encoding sHsp [13,14]. Some of these proteins, such as HspB1, HspB5, HspB6, and HspB8, are expressed ubiquitously [15,16] and are thoroughly analyzed [9,17,18,19,20]. The other members of the human sHsp family are tissue-specific and less investigated. One of these proteins, HspB7 (or cardiovascular Hsp, cvHsp), is predominantly expressed in insulin-dependent tissues, i.e., in heart, skeletal muscles, and adipocytes [15,21]. HspB7 seems to be involved in many significant intracellular processes. For instance, HspB7 participates in autophagy and prevents the aggregation of huntingtin fragments in the cell [22,23]. This protein is a highly effective electrophilic sensor [24] and, therefore, can participate in the protection of the cell from oxidative stress. HspB7 interacts with contractile and cytoskeletal proteins such as actin, filamin, and titin and can thus be involved in the regulation of heart and muscle development and contraction [21,25,26]. All these data were predominantly obtained on a cellular level, whereas the oligomeric structure and physicochemical properties of HspB7 were analyzed only superficially. This paper deals with the development of a simple and reliable method for recombinant human HspB7 purification, investigation of its quaternary structure, and ability to form hetero-oligomeric complexes with two other sHsps, namely, HspB6 and HspB8.

## 2. Results

### 2.1. Isolation of Untagged Recombinant HspB7

Upon starting our investigation, we were interested in developing a simple and reliable method of recombinant HspB7 isolation. The commonly used method of recombinant protein expression based on IPTG induction resulted in the accumulation of HspB7 in inclusion bodies. We attempted to isolate HspB7 from inclusion bodies using the earlier described method of Asthana et al. [27] and Mymrikov et al. [28]. Inclusion bodies were thoroughly washed and HspB7 was extracted with a solution containing 6 M of urea (see Materials and Methods). After stepwise dialysis, HspB7 was subjected to ion-exchange and size-exclusion chromatography (SEC) under nondenaturing condition. Thus, purified HspB7 formed multiple oligomers with different apparent molecular weights. All fractions obtained after SEC contained homogenous HspB7; however, these differently sized oligomers were very unstable and tended to irreversible aggregation. Therefore, we attempted to develop an alternative method of expression, decreasing accumulation of HspB7 in inclusion bodies and leaving a larger part of protein in a soluble fraction.

**Figure 1:**
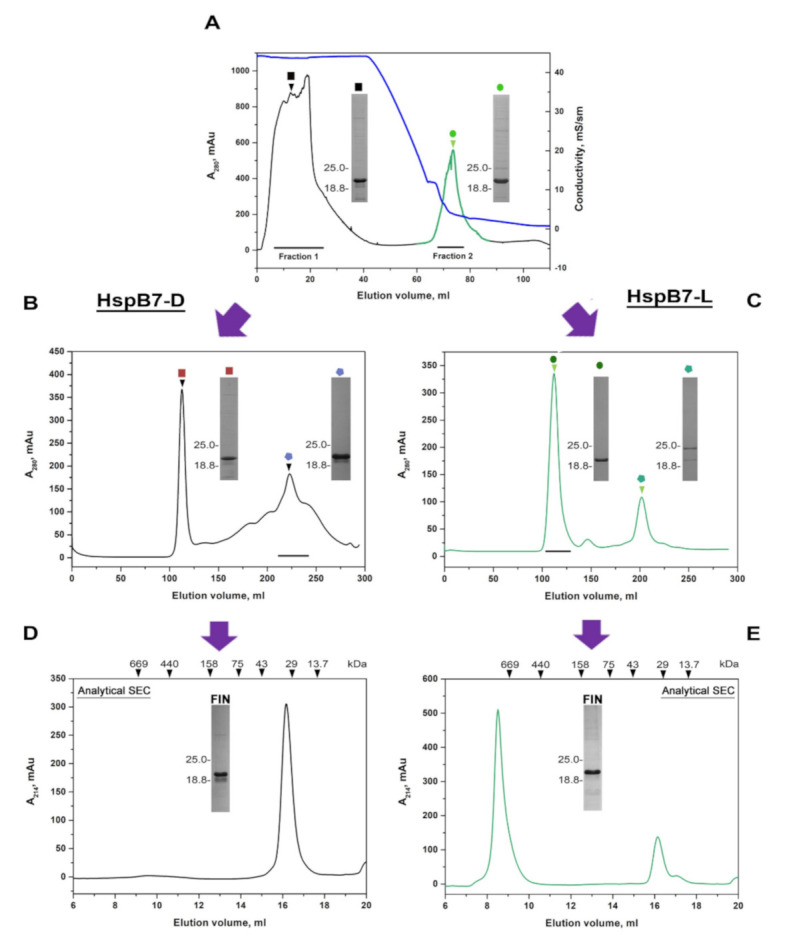
Chromatographic stages of recombinant HspB7 purification. (**A**) Hydrophobic chromatography of crude HspB7 preparation on HiTrap Phenyl column. Blue line represents conductivity. Fraction 1 and Fraction 2 were collected separately and subjected to SEC. (**B**) SEC of Fraction 1 loaded on preparative HiLoad Superdex 200. Fractions marked on the elution profile were collected, concentrated, and their oligomeric state was analyzed by analytical SEC on Superdex 200 column (panel **D**). (**C**) Proteins retarded on HiTrap Phenyl column (Fraction 2 in panel **A**) were pooled and subjected to SEC on HiLoad Superdex 200 column. Fractions containing proteins with a high molecular weight (marked on elution profile) were collected, concentrated, and subjected to analytical SEC of Superdex 200 column (panel **E**). Molecular weights (kDa) of protein standards are indicated at the top of panels (**D**,**E**), SDS-PAGE of fractions marked by colored squares and circles and final preparation (FIN) of HspB7 are presented on inserts.

Autoinduction performed in three-fold LB media at 20 °C resulted in predominant localization of HspB7 in a soluble fraction, although a significant part of HspB7 was again detected in inclusion bodies. HspB7 from the soluble fraction was purified by means of ammonium sulfate fractionation, followed by either ion-exchange or hydrophobic chromatography. In the case of hydrophobic chromatography, part of HspB7 was eluted in flow-through (Fraction 1) and another part (Fraction 2) was retarded on the column (Figure 1A). These fractions were collected separately and subjected to SEC on the Superdex 200 column. Two peaks were detected on the SEC of Fraction 1, unretarded on phenyl sepharose (Figure 1B). The first hypersharp peak was eluted in the exclusion volume, whereas the largest part of HspB7 was eluted in the broad peak with an apparent molecular weight in the range of 30–50 kDa (Figure 1B). Homogenous HspB7 eluted in this peak was concentrated and subjected to analytical SEC on the Superdex 200 Increase 10/30 GL column (Figure 1D) and only one peak with an apparent molecular weight of ~36 kDa was detected on the elution profile (Figure 1D). HspB7 retarded on phenyl sepharose column (Fraction 2 in Figure 1) was also subjected to preparative SEC on Superdex 200 (Figure 1C). Two peaks were detected on the elution profile. The first and largest peak was eluted close to the exclusion volume and had an apparent molecular weight of more than 600 kDa, and the considerably smaller peak had an apparent molecular weight in the range of 40–50 kDa (Figure 1C). The largest peak containing homogenous HspB7, was concentrated and subjected to analytical SEC on the Superdex 200 Increase 10/30 GL column, is shown in Figure 1E. Again, two peaks were detected on the elution profile. The first sharp peak was eluted close to the exclusion volume and had a molecular weight of more than 600 kDa; the second, much smaller peak had a molecular weight of ~36 kDa. Thus, recombinant HspB7 is present in two oligomeric forms: one form containing predominantly large oligomers with an apparent molecular weight of more than 600 kDa marked as HspB7-L (derived from Fraction 2 in Figure 1A) and another form containing only small oligomers (probably dimers) with an apparent molecular weight of ~36 kDa and marked as HspB7-D (derived from Fraction 1 in Figure 1A). Hydrophobic chromatography can be used for partial separation of these oligomeric forms and to obtain HspB7-D.

The data presented indicate that HspB7-D and HspB7-L differ in their binding to phenyl sepharose and, therefore, probably differ in their exposed hydrophobic surfaces. Indeed, titration of different species of HspB7 by hydrophobic probe bis-ANS revealed that HspB7-L has more exposed hydrophobic surfaces than HspB7-D (Supplementary Figure S1).

### 2.2. Oligomeric State of HspB7 and Its Dependence on the N-Terminal Domain and Oxidation

**Figure 2:**
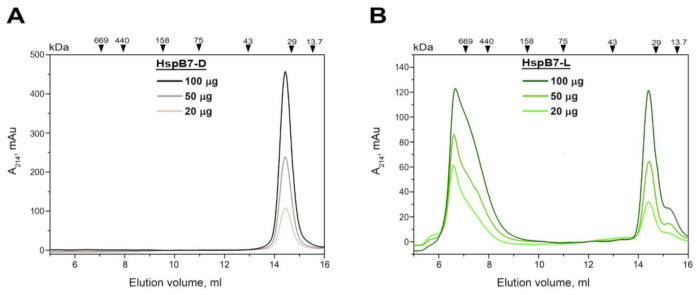
SEC of different quantities of HspB7-D (**A**) and HspB7-L (**B**) on the Superdex 200 Increase 10/30 GL column. 20, 50 or 100 μg of protein were loaded on the column. Elution volumes of protein standards and their molecular weights (kDa) are indicated above each panel.

Many sHsps form an equilibrium mixture of small and large oligomers [18]. As mentioned, recombinant HspB7 also forms two types of oligomers with apparent molecular weights of ~36 and more than 600 kDa. To analyze interconversion of these forms, we loaded different quantities of HspB7-D and HspB7-L on the Superdex 200 column. As indicated in Figure 2, a five-fold increase of protein loaded on the column was not accompanied by any significant changes in the oligomeric structure of both HspB7-D and HspB7-L. This probably indicates that interconversion of these oligomeric forms is particularly slow.

**Figure 3:**
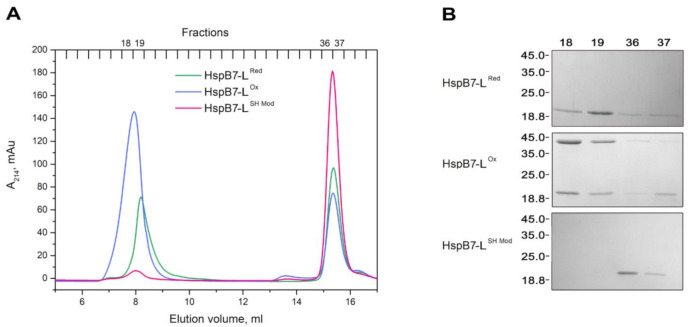
Oxidation and chemical modification of Cys residue affect oligomer structure of HspB7. (**A**) SEC of reduced (Red), oxidized (Ox), and modified by iodoacetamide (SH Mod) HspB7-L. (**B**) SDS-PAGE of high (fractions 18, 19) and low (fractions 36, 37) molecular weight oligomers under the nonreducing condition.

HspB7 contains the single Cys126 located at the end of β7 strand that is involved in the formation of inter-subunit interface of many sHsps [29]. Therefore, one can suppose that modification of Cys126 of HspB7 can somehow affect its oligomerization. To check this suggestion, we used HspB7-L obtained by ion-exchange chromatography coupled with SEC and containing both large and small oligomers. Mild oxidation of HspB7-L induced by overnight incubation at 37 °C and pH 8.0 increased the probability of large oligomer formation (Figure 3A). Mild oxidation was accompanied by the formation of HspB7 dimers crosslinked by disulfide bond (Figure 3B), although small quantities of crosslinked dimers were also detected in small oligomers with an apparent molecular weight of ~36 kDa (Figure 3B). Therefore, formation of a disulfide bond is not an indispensable condition for large oligomer formation; however, oxidation promotes the formation of large HspB7 oligomers. Effect of the single-SH group on oligomerization was also analyzed by means of covalent modification of Cys126 by iodoacetamide (Figure 3A). Modification of Cys126 of HspB7-L by iodoacetamide prevented the formation of large oligomers and HspB7 was presented only in the form of small oligomers with an apparent molecular weight of ~36 kDa.

**Figure 4:**
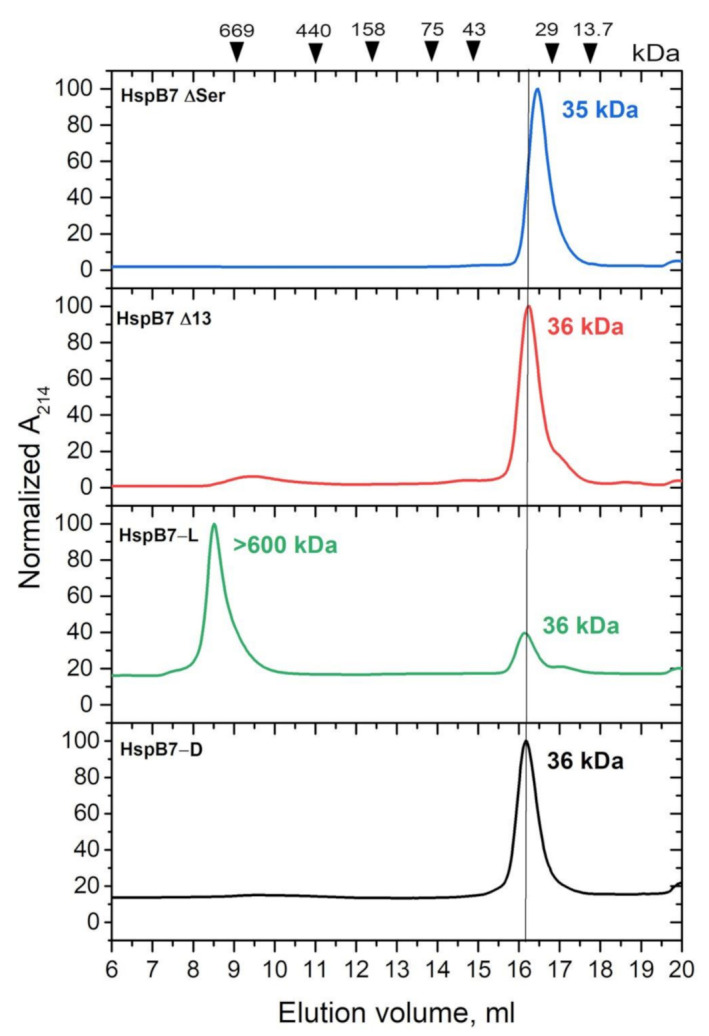
Normalized SEC elution profiles of the wild-type HspB7-D, HspB7-L, Δ13, and ΔSer mutants of HspB7. Elution volumes and apparent molecular weights of protein standards (kDa) are indicated at the top of the panel.

It is generally accepted that the N-terminal domain plays an important role in the formation of large oligomers of sHsp [8,12]. Therefore, we analyzed the effect of deletion of the first 13 residues (Δ13 mutant) and deletion of the unique polySer sequence located between residues 17 and 29 (ΔSer mutant) on the oligomeric state of HspB7 (Figure 4). As already mentioned, depending on the isolation procedure, the wild-type HspB7 is present in the form of dimers (HspB7-D) and large (HspB7-L) oligomers with apparent molecular weights of ~36 and more than 600 kDa, respectively (Figure 4). HspB7-D weakly binds to phenyl sepharose and its surface hydrophobicity measured by the hydrophobic probe bis-ANS is lower than that of HspB7-L (Supplementary Figure S1). The ΔSer mutant of HspB7, forming only small oligomers has even less surface hydrophobic sites than HspB7-D and is not retarded on phenyl sepharose (Supplementary Figures S1 and S2). Independent of the isolation procedure, both Δ13 and ΔSer mutants form only small molecular weight oligomers with apparent molecular weights of ~36 and 35 kDa. Thus, the N-terminal domain affects the oligomerization of HspB7.

### 2.3. Interaction of HspB7 with HspB6 and HspB8

With the similar organization of their primary structure, small heat shock proteins can form hetero-oligomeric complexes [30,31]. Therefore, it seemed reasonable to analyze the interaction of HspB7 with the two other ubiquitously expressed sHsps: HspB6 and HspB8. For this purpose, we used the previously described method based on mild crosslinking of Cys residues located in the β7 strand involved in the inter-subunit interaction [29,32,33]. In these experiments, we used the wild-type HspB7 having the single Cys residues (Cys126) located at the C-terminal end of the β7 strand and the so-called Cys-mutants of HspB6 and HspB8. The Cys-mutant of HspB6 carried two mutations C46S/E116C, and the Cys-mutant of HspB8 carried four mutations C10S/C99S/C195S/N138C. These mutations had no effect on the structure of sHsp and had only one Cys residue located in the position homologous to Cys137 of HspB1, i.e., in the interface of sHsp monomers [29].

**Figure 5:**
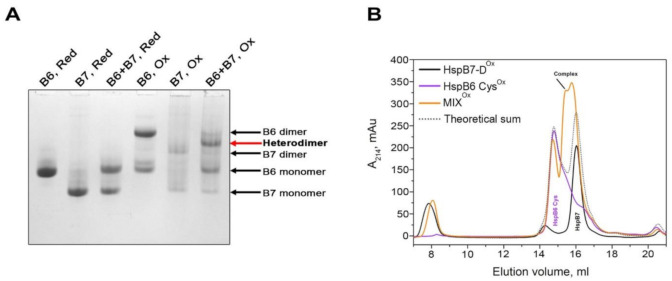
Interaction of HspB6 and HspB7. (**A**) Urea gel electrophoresis of the reduced Cys-mutant of HspB6 (B6, Red), reduced B7 (B7, Red), equimolar mixture of the reduced Cys-mutant of HspB6 and HspB7 (B6+B7, Red), oxidized Cys-mutant of HspB6 (B6, Ox), oxidized HspB7 (B7, Ox), and equimolar mixture of the oxidized Cys-mutant of HspB6 and HspB7 (B6+B7, Ox). Positions of monomers and dimers of HspB6 and HspB7 are marked by black arrows; position of the crosslinked HspB7/HspB6 complex is marked by red arrow. (**B**) SEC of oxidized HspB7 (HspB7-D^Ox^, black), oxidized Cys-mutants of HspB6 (HspB6 Cys^Ox^, violet), oxidized equimolar mixture of Cys-mutant of HspB6 and HspB7 (MIX^Ox^, orange), and arithmetic sum of elution profiles of isolated oxidized HspB6 and HspB7 (Theoretical sum, grey dotted line).

Both HspB6 and HspB7 are highly expressed in the heart [21,34]; therefore, it is reasonable to analyze the interaction of these two members of the sHsp family. Both proteins were reduced by an excess of DTT and afterwards dialyzed separately or in an equimolar mixture overnight. Thus, obtained samples were analyzed by means of electrophoresis and SEC. Molecular weights and pI values of HspB6 and HspB7 are very similar, and this complicates the investigation of their hetero-oligomeric complex formation. However, utilizing urea-PAGE, we were able to detect the formation of a new band, formed after disulfide crosslinking of HspB6 and HspB7 (Figure 5A). The formation of this band was accompanied by a decrease in the intensity of the bands corresponding to HspB6 and HspB7 monomers and dimers (Figure 5A), and the reduction resulted in an increase in the intensity of the HspB6 and HspB7 monomer bands. This indicates that these new bands correspond to the crosslinked HspB6–HspB7 complex.

Oxidized HspB6 was eluted as a broad peak with a maximum corresponding to the apparent molecular weight of 54 kDa (violet line in Figure 5В). Oxidized HspB7 was eluted in two peaks with apparent molecular weights of more than 600 kDa and 36 kDa (black line in Figure 5B). The crosslinked complex of HspB6 and HspB7 was eluted in the form of multiple peaks with maximums corresponding to protein species with apparent molecular weights of 39–44 kDa (orange line in Figure 5B). The elution profile of the crosslinked mixture of HspB6 and HspB7 (orange line in Figure 5B) is different to that obtained by the arithmetic summation of elution profiles of isolated HspB6 and HspB7 (grey dotted line in Figure 5B).

**Figure 6:**
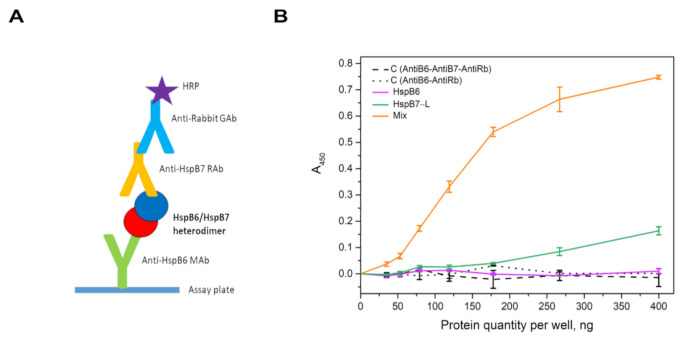
(**A**) Detection of HspB6 and HspB7 hetero-oligomers by means of sandwich ELISA method. Immunological plate was coated with monoclonal mouse anti-HspB6 antibodies (MAb) used as a capture antibody. Rabbit polyclonal monospecific anti-HspB7 antibodies (RAb) recognized only HspB7 retarded on the plate due to its interaction with HspB6. Anti-HspB7 antibodies were detected by HRP-conjugated goat anti-rabbit immunoglobulin antibodies (GAb). (**B**) Isolated HspB6 (magenta), isolated HspB7 (green) or equimolar mixture of HspB6 and HspB7 (orange) were added to mouse anti-HspB6 antibody immobilized on the immunological plate. HspB7 was detected by rabbit anti-HspB7 antibodies followed by HRP-conjugated goat anti-rabbit antibodies. In control experiments, immobilized anti-HspB6 antibodies were incubated either with anti-HspB7 antibodies + HRP-conjugated anti-rabbit antibodies (dashed line) or only with HRP-conjugated anti-rabbit antibodies (dotted line). Data presented are means of three independent experiments ± SD.

The similarities in molecular weights and isoelectric points of HspB6 and HspB7 complicate the investigation of their interaction. To overcome these difficulties, we used a slightly modified version of the earlier described immunochemical approach [35,36]. Immunological plate was coated by monoclonal mouse anti-HspB6 antibodies (MAb) and incubated with isolated HspB6, isolated HspB7, or an equimolar mixture of HspB6 and HspB7. The hetero-oligomeric complex of HspB6 and HspB7 was detected by polyclonal monospecific rabbit anti-HspB7 antibodies (RAb) followed by HRP-conjugated goat antirabbit immunoglobulin antibodies (GAb) (Figure 6A). As expected, anti-HspB7 antibodies did not cross-react with HspB6 bound to anti-HspB6 antibodies (magenta line in Figure 6B). Anti-HspB7 antibodies were practically unable to detect HspB7 if it was added to the immunological plate in an isolated form (green line in Figure 6B). However, anti-HspB7 antibodies detected HspB7 if an equimolar mixture of HspB6 and HspB7 was added to immobilized anti-HspB6 antibodies (orange line in Figure 6B). These data indicate that HspB7 was retarded on the plate only due to the formation of the hetero-oligomeric complex with HspB6 that interacted with immobilized anti-HspB6 antibodies.

**Figure 7:**
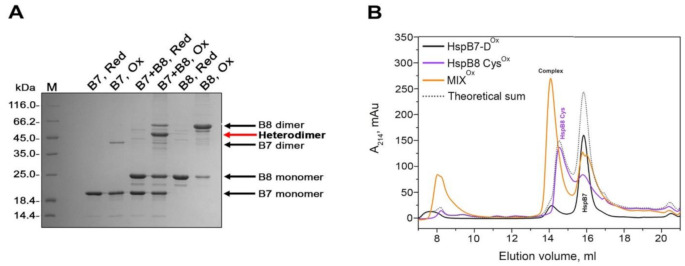
Interaction of HspB7 and HspB8. (**A**) SDS-PAGE of reduced HspB7 (B7, Red), oxidized HspB7 (B7, Ox), reduced mixture of HspB7 and Cys-mutant of HspB8 (B7 + B8, Red), of oxidized mixture of HspB7 and Cys-mutant of HspB8 (B7 + B8, Ox), reduced and oxidized Cys-mutant of HspB8 (B8, Red and B8, Ox). Positions of monomers and dimers of HspB7 and HspB8 are marked by black arrows, position of crosslinked HspB7-HspB8 complex is marked by red arrow. (**B**) SEC of oxidized HspB7 (HspB7-D^Ox^, black), oxidized Cys-mutants of HspB8 (HspB8 Cys^Ox^, violet), oxidized equimolar mixture of Cys-mutant of HspB8 and HspB7 (MIX^Ox^, orange), and arithmetic sum of elution profiles of isolated oxidized HspB7 and HspB8 (Theoretical sum, grey dotted line).

Both HspB7 and HspB8 are expressed in the same tissues (heart and skeletal muscles). Therefore, it seems reasonable to analyze the interaction of these two sHsps. In this case, we used the earlier described approach based on the utilization of Cys-mutants of HspB8. The apparent molecular weights of HspB7 and HspB8 are significantly different; therefore, we were able to use SDS-PAGE for the detection of crosslinked heterodimer complexes (Figure 7A).

Depending on the conditions, HspB7 can undergo disulfide crosslinking and formation of a crosslinked dimer (Figure 7A). Such crosslinking is strongly dependent on conditions and, as a rule, is less effective than in the case of Cys-mutants of HspB8 (Figure 7A). After oxidation of the equimolar mixture of HspB7 and HspB8, we detected a new band with an apparent molecular weight of 45 kDa (marked by red arrow). Formation of this band was accompanied by a decrease in the bands corresponding to HspB7 and HspB8 monomers and dimers. Reduction resulted in the disappearance of this band and accumulation of HspB7 and HspB8 monomers. Moreover, the apparent molecular weight of this new band is close to the sum of the apparent molecular weights of HspB7 (~19 kDa) and HspB8 (~25 kDa) and equal to 44 kDa, thus indicating that this new band corresponds to the disulfide-crosslinked HspB7–HspB8 complex.

Oxidized HspB7 and HspB8 and their mixture were subjected to SEC (Figure 7B). Oxidized HspB7 migrated in the form of two peaks with apparent molecular weights of more than 600 kDa and 36 kDa (black line in Figure 7B). Oxidized HspB8 produced two peaks with apparent molecular weights of 34 and 62 kDa (violet line in Figure 7B). Oxidized equimolar mixture of HspB7 and HspB8 was eluted in the form of multiple peaks with apparent molecular weights of more than 600, 79 and 36 kDa (orange line in Figure 7B). It is worthwhile mentioning that the elution profile of the crosslinked mixture of HspB7 and HspB8 is different to that of the arithmetic sum of elution profiles of isolated oxidized HspB7 and HspB8 (grey dotted line in Figure 7B).

## 3. Discussion

Described in 1999 [21], HspB7 remains one of the less-studied proteins of the sHsp family. The largest part of investigations dealing with this protein were performed on a cellular level, whereas physicochemical and biochemical properties remain poorly characterized. This is probably explained by certain difficulties in the isolation of this protein. The first attempts to isolate untagged rat recombinant HspB7 resulted in the homogenous protein that formed two types of oligomers with apparent molecular weights of ~40 and more than 200 kDa [37]. Mouse His-tagged HspB7 was isolated by metal-affinity chromatography [38]. Quaternary structure of this protein was not characterized; however, it was completely sedimented after centrifugation at 150,000× *g* for 90 min. Finally, attempts to isolate HspB7 from inclusion bodies by using extraction with high urea concentrations resulted in obtaining protein forming multiple oligomers on SEC [28]. All these data mean that isolated recombinant protein tends to form either considerably large oligomers or aggregates in the course of isolation or storage. It should be pointed out that in extracts of HEK293 cells, HspB7 was presented only in the form of small oligomers (probably dimers) [22,23]. Therefore, we tried to develop a simple and effective method for the isolation of small and large oligomers of HspB7 and to analyze factors that are responsible for the formation of large oligomers.

**Figure 8:**
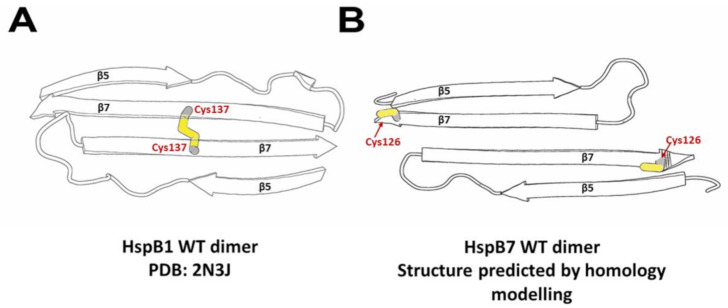
Diagram of ACD interface and cysteine location in two sHsps. (**A**) HspB1-oxidized dimer PDB 2N3J. (**B**) Model of HspB7 homodimer constructed by the SWISS-MODEL server with PDB 4JUS as a template. Only β5 and β7 strands of sHsps monomers are shown.

Our attempts to isolate HspB7 from inclusion bodies were marginally successful; therefore, we decided to isolate this protein from a soluble fraction. By varying the expression condition and *E. coli* strains, we found that HspB7 solubility was dependent on temperature and conditions of induction. We found that autoinduction of HspB7 in the Rosetta2 (DE3) pLysS strain at decreased temperature resulted in the accumulation of significant quantities of this protein in a soluble fraction. During hydrophobic chromatography, part of HspB7 was eluted in the flow-through, whereas another part of HspB7 was retarded on phenyl sepharose (Figure 1A). Further purification of HspB7 unbound on phenyl sepharose resulted in obtaining small oligomers with apparent molecular weights of ~36 kDa, which probably correspond to the HspB7 dimer (calculated molecular weight of HspB7 monomer is equal to 18.6 kDa) (Figure 1D). Purification of HspB7 retarded on phenyl sepharose resulted in obtaining HspB7 predominantly containing large oligomers with apparent molecular weights of more than 600 kDa and small quantities of oligomers with apparent molecular weights of ~36 kDa (Figure 1E). Equilibration between large and small oligomers seems to be slow, and formation of large oligomers is not practically dependent on protein concentration (Figure 2). We tried to find factors affecting the oligomeric state of HspB7. Mild oxidation led to the accumulation of large oligomers at the expense of small HspB7 oligomers (Figure 3). Moreover, oxidation was accompanied by the formation of disulfide-crosslinked dimers (Figure 3); this finding is rather unexpected. Indeed, if the structure of HspB7 ACD is similar to that of other sHsps, then Cys126 located at the end of the β7 strand of one monomer is far from Cys126 located in the antiparallel β7 strand of another monomer (Figure 8). Although the efficiency of HspB7 disulfide crosslinking is lower than in the case of HspB1 or Cys-mutants of other sHsps [29], crosslinking Cys126 of HspB7 means that either this residue is very reactive [24] or that the interface of two HspB7 monomers is different to the corresponding interface of other sHsps.

Three orientations of β7 strands were postulated in the literature [39]. In these orientations (the so-called AP_I_, AP_II_, and AP_III_), the β7 strands of two neighboring monomers are slightly shifted across each other. Shifting of these strands can increase the probability of Cys oxidation and formation of the disulfide bond. Formation of the disulfide bond can affect HspB7 structure and affect the formation of large oligomers. Probable involvement of Cys126 in HspB7 oligomerization was confirmed in experiments where modification of this residue with iodoacetamide prevented the formation of large oligomers (Figure 3).

The data of literature indicate that the N-terminal domain plays an important role in the formation of large oligomers of sHsps [40,41]. To check whether the N-terminal part also participates in the formation of large HspB7 oligomers, we obtained two deletion mutants leaking either the first 13 amino acid residues or the so-called poly-Ser sequence (residues 17–29) that is unique for this protein. Both Δ13 and ΔSer mutants formed only small oligomers with apparent molecular weights of 32–36 kDa and were unable to form large oligomers, thus indicating involvement of the NTD in the oligomerization (or aggregation) of HspB7. In this respect, it is worthwhile to mention that the NTD plays a crucial role in HspB7-induced suppression of polyQ protein aggregation [22,23]. Thus, the NTD plays dual roles, somehow affecting the association (aggregation) of HspB7 and simultaneously affecting its antiaggregation activity. By varying conditions of expression and purification, modifying the single-SH group or truncating certain parts of the NTD, we obtained recombinant HspB7 species forming only small oligomers that were detected earlier in extracts of HEK293 cells [22,23] and that can be useful for further investigation of properties and chaperone-like activity of this protein.

Many sHsps form hetero-oligomeric complexes [30,31] and this affects the oligomeric structure and certain properties of these proteins [42]. The data on HspB7 hetero-oligomerization are contradictory. On one site, it was postulated that HspB7 interacts with HspB8 [43], whereas the other results [23] indicate that HspB7 is unable to form hetero-oligomers with other sHsps. We attempted to analyze the interaction of HspB7 with HspB6 and HspB8, two other sHsps ubiquitously expressed in practically all human tissues. HspB6 and HspB7 have very similar molecular weights and pI values, and this complicates the detection of hetero-oligomer formation. However, by using disulfide crosslinking and urea gel electrophoresis, we were able to detect a new band corresponding to the crosslinked HspB6–HspB7 complex (Figure 5A). These data were confirmed using SEC. Indeed, the elution profile of the oxidized mixture of HspB7 and the Cys-mutant of HspB6 is different to the arithmetic sum of elution profiles of isolated HspB6 and HspB7 (Figure 5B), thus indicating that proteins with apparent molecular weights of 39–44 kDa eluted between the oxidized Cys-mutant of HspB6 and HspB7 correspond to the covalent crosslinked complex of HspB6–HspB7. These results were confirmed in immunochemical experiments (Figure 6). In this case, HspB7 was retarded on an immunological plate coated with anti-HspB6 antibodies only if it was added in the form of an equimolar mixture with HspB6.

We also analyzed the interaction of HspB7 and HspB8 using the Cys-mutant of HspB8. The molecular weights of HspB7 and HspB8 are different; therefore, we were able to detect the formation of the crosslinked hetero-oligomeric complex by SDS-PAGE (Figure 7A). Disulfide crosslinking of the wild-type HspB7 and Cys-mutant of HspB8 resulted in accumulation of the protein band with an apparent molecular weight of 44 kDa, corresponding well to the sum of the apparent molecular weights of HspB7 and HspB8 (Figure 7A). These results were confirmed by SEC. The elution profile of the crosslinked HspB7–HspB8 complex contains protein peaks with apparent molecular weights of 79–80 kDa that were absent on the elution profiles of oxidized HspB7 and of the Cys-mutant HspB8 (Figure 7B). The data of SDS-PAGE indicate that this peak indeed contains the crosslinked complex of HspB7 and HspB8 (data not shown). Thus, the data presented indicate that HspB7 can form disulfide-crosslinked hetero-oligomeric complexes with HspB6 and HspB8. Formation of these complexes seems to be rather specific, since HspB7 was unable to form disulfide-crosslinked hetero-oligomeric complexes with HspB1 (data not shown). In certain tissues (such as the heart and muscles), the concentration of HspB7 and its potential partners, HspB6 and HspB8, is very high [44]. This increases the probability of heterocomplex formation. Hetero-oligomerization can affect cellular location, chaperone-like activity, protection against oxidative stress, and interaction of sHsps with the cytoskeleton and contractile apparatus. This hypothesis requires further detailed experimental verification.

## 4. Materials and Methods

### 4.1. Cloning of the Wild-Type Proteins and Their Mutants

The plasmid-containing full copy of human HspB7 was obtained from Addgene (Watertown, MA, USA) #63108 and recloned into the pET23b(+) plasmid at *NdeI* and *XhoI* sites. This plasmid was used as a template to produce deletion mutants of HspB7 lacking the first 13 residues (the so-called Δ13 mutant) by PCR with forward primer (5′-GATCCATATGAGTTTCCATTCCTCTTCTTCTTC-3′) and reverse primer (5′-ATGCTAGTTATTGCTCAG-3′). A mutant-lacking poly Ser sequence (residues 17–29, the so-called ΔSer mutant) was obtained by means of Quick-change mutagenesis with forward primer (5′-GAGAGAAGTTTCCATGCCTCCCGTGCCCTCCCGGCCCAGGA-3′) and reverse primer (5′-GAGGCATGGAAACTTCTCTCCGCTCGGAAGGTGGAAGAGGTTCTGTG-3′). All constructs were cloned into the pET23b(+) vector, integrity and lack of additional mutations were confirmed by DNA sequencing. Plasmids containing full-size wild-type HspB6 and HspB8 genes were described earlier [29]. Plasmids containing the sequence coding the so-called Cys-mutant of HspB6 with double mutation C46S/E116C and Cys-mutant of HspB8 with four-fold mutations C10S/C99S/C195S/N138C were also described earlier [29].

### 4.2. Expression and Purification of Recombinant Proteins

Expression and purification of the wild-type HspB6, HspB8, and its Cys-mutants were performed as described earlier [29,45,46]. HspB7 and its deletion mutants were expressed in the Rosetta2 (DE3) pLysS strain of *E. coli* under different conditions. In the first case, bacteria were grown at 37 °C up to the OD of 0.6–0.8 at 600 nm; expression was induced by the addition of 0.5 mM IPTG and lasted for 6 h at 37 °C. Under these conditions, the largest part of recombinant HspB7 accumulated in inclusion bodies. In the second and third cases, bacteria were grown in three-fold LB media at 37 °C for 8 h and subsequently incubated overnight at either 20 or 30 °C. Under these conditions, the largest part of recombinant protein was detected in the soluble fraction, although a significant part of HspB7 moved to inclusion bodies. Better results were obtained if overnight incubation was performed at 20 °C.

We attempted to isolate HspB7 both from inclusion bodies and the soluble fraction. In the first case, we used the method described by Asthana et al. [27]. Inclusion bodies were thoroughly washed in buffer A (20 mM phosphate pH 7.4, 100 mM NaCl, 2 mM DTT and 0.1 mM PMSF) containing 0.05% Triton X-100. HspB7 was extracted with buffer A containing 6 M urea. After stepwise dilution and dialysis, HspB7 was subjected to ion-exchange chromatography on a 5-mL HiTrapQ column equilibrated with buffer B (20 mM Tris-acetate pH 7.6, 10 mM NaCl, 0.1 mM EDTA, 0.1 mM PMSF and 15 mM ME (β-mercaptoethanol)) and, after washing, eluted with linear gradient (10–360 mM) of NaCl. Ion-exchange chromatography was followed by size-exclusion chromatography on a HiLoad Superdex 200 prep column equilibrated with buffer C (20 mM Tris-acetate pH 8.0, 150 mM NaCl, 0.1 mM EDTA, 0.1 mM PMSF and 2 mM DTT). Under these conditions, HspB7 was eluted in the form of multiple peaks, demonstrating a tendency to form different size oligomers. This method provided a homogeneous protein presented in multiple heterogeneous oligomer forms that were very unstable and precipitated under storage.

Two methods were used for the purification of HspB7 from the soluble fraction. Bacteria suspended in lysis buffer (50 mM Tris-HCl pH 8.0, containing 150 mM NaCl, 0.1 mM PMSF, 0.1 mM EDTA and 2 mM DTT) were subjected to lysozyme treatment and sonicated. The obtained extract was subjected to ammonium sulfate fractionation in the range of 0–30%. The pellet was dissolved and subjected either to ion-exchange or hydrophobic chromatography. In the first case, the protein obtained after ammonium sulfate fractionation was dissolved and dialyzed against buffer B (20 mM Tris-acetate pH 8.0, containing 10 mM NaCl, 0.1 mM EDTA, 0.1 mM PMSF and 2 mM DTT). The protein was loaded on a 5-mL HiTrapQ column equilibrated with buffer B and, after washing, eluted with linear gradient (10–360 mM) of NaCl. In the final stage, HspB7 was subjected to size-exclusion chromatography on HiLoad Superdex 200 prep column equilibrated with buffer C (20 mM Tris-acetate pH 8.0, containing 150 mM NaCl, 0.1 mM EDTA, 0.1 mM PMSF and 2 mM DTT), aliquoted and stored at −20 °C. In the second case, the pellet obtained after ammonium sulfate fractionation was dissolved in buffer D (20 mM Tris-acetate pH 8.0, 0.1 mM EDTA, 0.1 mM PMSF and 2 mM DTT) and centrifuged (12.000× *g*, 15 min). Ammonium sulfate was added up to 0.3 M and protein was loaded on a 5-mL HiTrap Phenyl column equilibrated with buffer D containing 0.3 M of ammonium sulfate. After washing, the column was developed with 5 column volumes of 0–95% of buffer D followed by 7 column volumes of 95–100% of buffer D. HspB7 eluted in flow through fractions or adsorbed on the column was collected separately, concentrated and subjected to size-exclusion chromatography on a High-Load Superdex 200 prep column equilibrated with buffer C. Purified protein was concentrated, dialyzed against buffer B, aliquoted and stored at −20 °C. The HspB7 concentration was measured utilizing the Bradford assay [47] with BSA as a standard.

### 4.3. Size-Exclusion Chromatography

Analytical size-exclusion chromatography (SEC) was performed on a Superdex 200 Increase 10/30 GL column equilibrated with buffer E (50 mM phosphate buffer pH 7.4, containing 150 mM NaCl) either in the presence or absence of 2 mM DTT. Since HspB7 does not contain Trp and has only one Tyr residue, it has very low absorbance at 280 nm (ε_280_ 0.1% = 0.08); therefore, the elution profile was recorded at 214 nm. The column was calibrated with protein standards thyroglobulin (669 kDa), ferritin (440 kDa), aldolase (158 kDa), conalbumin (75 kDa), ovalbumin (43 kDa), carboanhydrase (29 kDa), and RNAse (13.7 kDa). The sample volume was equal to or less than 100 µL and the rate of elution was equal to 0.75 mL/min.

### 4.4. Modification of SH-Group of HspB7

The single-SH group of HspB7 (Cys126) was subjected to mild oxidation or modification with iodoacetamide. In the first case, mild oxidation occurs with atmospheric oxygen during prolonged (overnight) dialysis of HspB7 (2.5 mg/mL) against 50 mM phosphate (pH 8.0) containing 150 mM NaCl and 0.1 mM PMSF. Afterwards, 100 µL of unoxidized and oxidized proteins were subjected to SEC on the Superdex Increase GL 10/300 column. In the second case, HspB7 (0.6 mg/mL) in 50 mM phosphate (pH 7.6) containing 150 mM NaCl and 0.1 mM PMSF was incubated for 1 h with 3-mM DTT at 42 °C. Iodoacetamide was added up to a final concentration of 8 mM and incubation lasted for an additional 1 h at 37 °C. The samples of modified and unmodified proteins were subjected to SEC.

### 4.5. Interaction of HspB7 with Hydrophobic Probe Bis-ANS

Exposed hydrophobic surfaces of HspB1, HspB7-D, HspB7-L, and ∆Ser mutant were analyzed using the fluorescent probe bis-ANS. All the experiments were performed on a CaryEclipse (Varian) spectrofluorometer in a thermostated cell (30 °C) in buffer F (50 mM potassium phosphate pH 7.5, 150 mM NaCl, 0.1 mM EDTA and 15 mM ME). Protein samples (0.03 mg/mL, equal to 1.7 µM per HspB7 monomer) were titrated with a stock solution of bis-ANS, so that the total concentration of the fluorescent probe was in the range of 1–7 µM. Fluorescence was excited at 385 nm (slit width 5 nm) and recorded at 495 nm (slit width 5 nm).

### 4.6. Crosslinking of HspB7 and Cys-Mutants of HspB6 and HspB8

Isolated HspB7 or isolated Cys-mutants were incubated for 1 h in buffer C at 37 °C with 15 mM DTT to reduce all SH groups. Isolated proteins or their equimolar mixture with the final concentration of each protein equal to 15 µM were incubated for 1 h at 42 °C. These conditions provided subunit exchange and facilitated the formation of hetero-oligomers. All protein samples were dialyzed overnight at 37 °C against the buffer containing 50 mM Tris-HCl pH 8.0, 50 mM KCl, and 0.1 mM PMSF. Protein composition of samples was determined by means of either SDS-PAGE [48] (for complexes formed by HspB7 and Cys-mutant of HspB8) or urea gel electrophoresis (for complexes formed by HspB7 and Cys-mutant of HspB6) performed with the system of Reisfeld et al. [49] or Davis [50]. Aliquots of samples were loaded on the Superdex 200 Increase GL 10/300 column and subjected to SEC. Fractions (400 μL) were collected and proteins were precipitated by addition of TCA (final concentration 10%). The protein pellet was washed twice by acetone, dried, and subjected to electrophoresis.

### 4.7. ELISA Experiments

A high-binding, 96-well immunological plate was coated with mouse monoclonal anti-HspB6 antibodies (clone 20-6, 400 ng per well), kindly provided by Prof. Alexey Katrukha (Lomonosov Moscow State University, Moscow, Russia). The plate was blocked with TBST (0.1% Tween-20 in PBS) and incubated with different quantities of isolated HspB6 (30 µM), isolated HspB7 (30 µM), or an equimolar mixture of two proteins (60 µM total). The quantity of each protein loaded in the well varied between 25 and 400 ng. After 1 h incubation, the wells were washed three times with PBS. Afterwards, we added polyclonal monospecific rabbit anti-HspB7 antibodies (100 ng per well), kindly provided by Dr. Natalya Tikhomirova (Lomonosov Moscow State University, Moscow, Russia). The plate was incubated for 30 min, washed three times with PBS and commercial HRP-conjugated anti-rabbit immunoglobulin antibodies (Abcam, ab6721) in TBST were added and incubated for 30 min. The plate was washed six times with PBS. Finally, substrates (tetramethylbenzidine and hydrogen peroxide in 0.1 M citrate buffer pH 4.0) were added and the absorbance was read using the microplate reader (Victor, Perkin Elmer) at 450 nm.

## Supplementary Materials

The following are available online at https://www.mdpi.com/article/10.3390/ijms22157777/s1.

## Abbreviations

ACD	α-crystallin domain
CTD	C-terminal domain
DTT	dithiotreitol
IPTG	isopropyl β-D-1-thiogalactopyranoside
ME	β-mercaptoethanol
NTD	N-terminal domain
PMSF	phenylmethanesulfonyl fluoride
SDS	sodium dodecyl sulfate
SEC	size-exclusion chromatography
sHsp	small heat shock proteins
