# Supplementary material for: Quaternary Structure and Hetero-Oligomerization of Recombinant Human Small Heat Shock Protein HspB7 (cvHsp)
Source: Int J Mol Sci. 2021 Jul 21;22(15):7777. doi: 10.3390/ijms22157777 (PMC8345930; doi:10.3390/ijms22157777)
Supplement: Supplementary file 1 [file ijms-22-07777-s001.zip › ijms-1294878-supplementary.pdf]

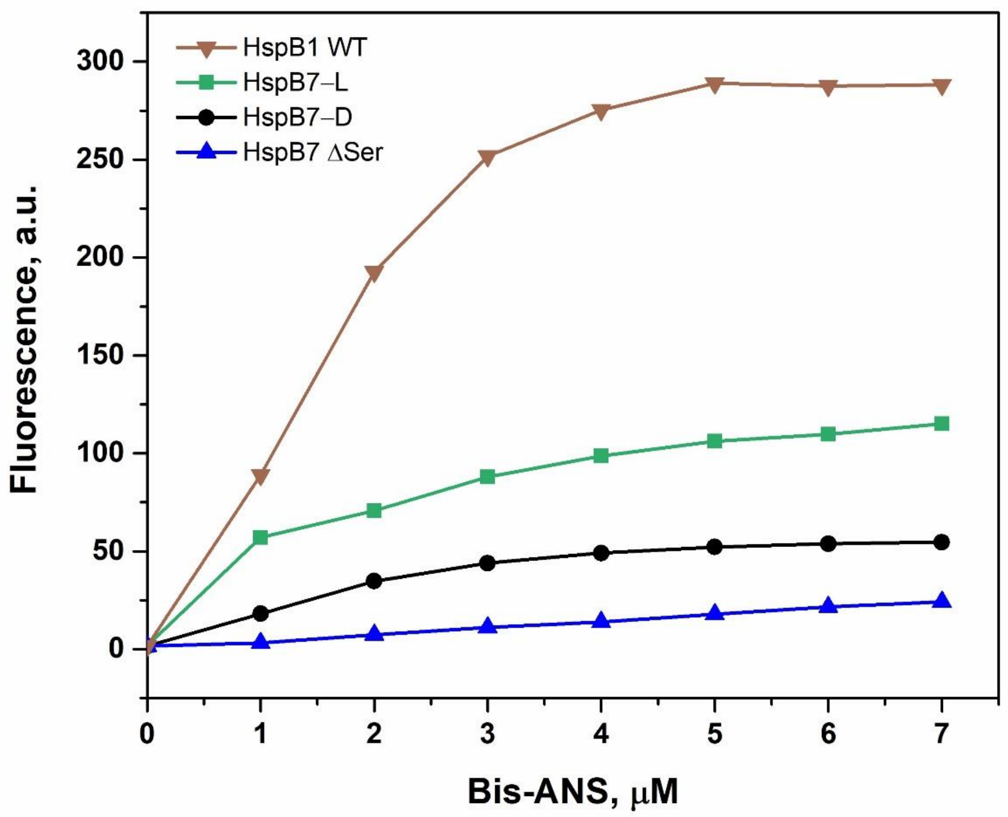

Supplement Figure S1. Surface hydrophobicity of HspB1 (brown), HspB7-L (green), HspB7-D (black) and ΔSer mutant of HspB7 (blue) studied by fluorescence probe bis-ANS. The wild type HspB1 was used as positive standard. Fluorescence was excited at 385 and recorded at 495 nm.

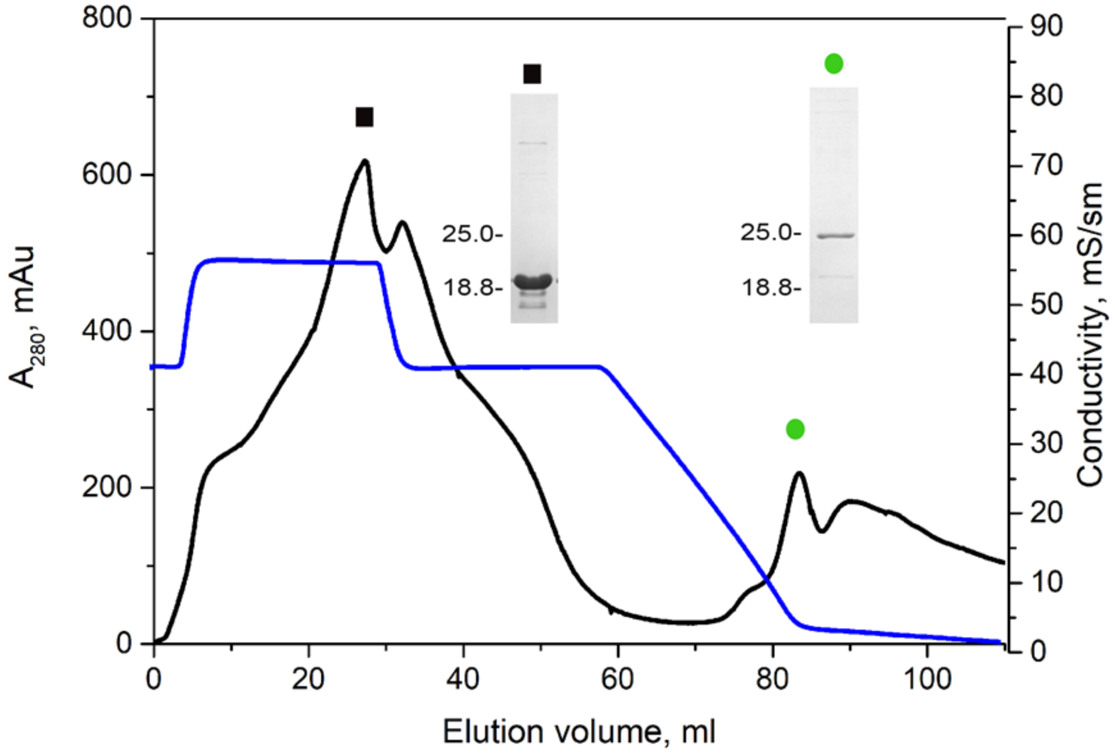

Supplement Figure S2. Chromatography of ΔSer mutant on HiTrap Phenyl-Sepharose. Crude preparation of ΔSer mutant of HspB7 was loaded on Phenyl-Sepharose and eluted by decreasing gradient of ammonium sulfate (blue line). SDS-PAGE of fractions marked by colored squares and circles are shown on inserts.
